# Diffuse Reflectance-Based Femtosecond Stimulated Raman Spectroscopy of Opaque Suspensions

**DOI:** 10.1021/acs.analchem.3c02491

**Published:** 2023-10-18

**Authors:** Steven A. Diaz, David W. McCamant

**Affiliations:** University of Rochester, Department of Chemistry Rochester, New York, New York 14534, United States

## Abstract

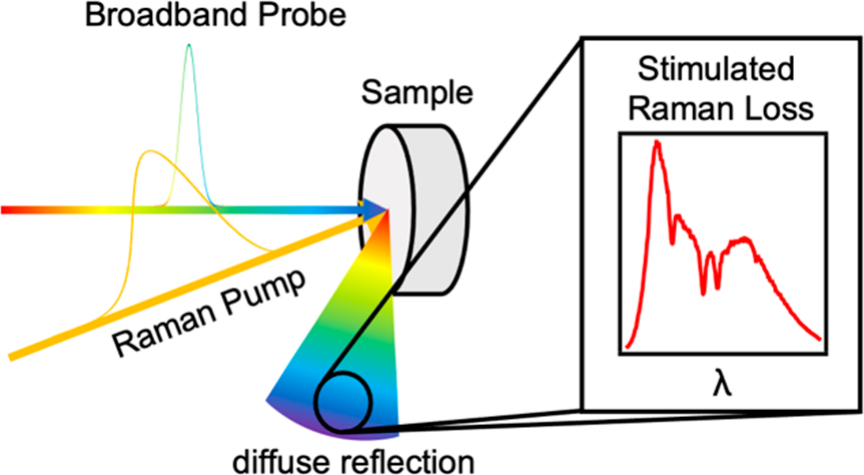

By augmentation of the collection optics utilized in transmission-based femtosecond stimulated Raman spectroscopy (FSRS), two novel diffuse reflectance-based femtosecond stimulated Raman spectroscopy (drFSRS) techniques were developed. These techniques were then used to collect the Raman spectra of opaque systems, those being cyclohexane-intercalated poly(tetrafluoroethylene) microbeads and ethanol in 1% intralipid solutions. The resulting drFSRS data from the cyclohexane:PTFE system show significant distortion of the depolarization ratio of the 803 cm^–1^ cyclohexane peak, indicating a loss of incident pump:probe polarization in a scattering environment. The drFSRS data from the ethanol in 1% intralipid solution demonstrate less signal strength but equal spectral resolution when compared to transmission-based FSRS of the same sample. The results presented in this Technical Note demonstrate the current capabilities of collecting stimulated Raman spectra of opaque systems using drFSRS.

Femtosecond stimulated Raman spectroscopy (FSRS) has demonstrated its ability to reveal complex molecular dynamics. From tracking changes in molecular structure at subpicosecond resolution^1,2^ to capturing resonance Raman cross sections of fluorescent dyes,^3^ the unique advantage of fluorescence-free measurements that FSRS offers grants chemists the ability to discern structural dynamics and function under conditions typically used for photochemistry that are inaccessible to traditional, spontaneous Raman spectroscopy.

Conventional “spontaneous” Raman spectroscopy offers the advantageous ability to probe opaque nontransmissive samples, a feature elusive to traditional FSRS. In comparison, nearly all stimulated Raman spectroscopy (SRS) methods depend on detecting the transmitted beam and therefore require transparent samples. Despite this limitation, SRS microscopy has been successfully deployed for biomedical imaging, but it typically depends on generating imaging contrast using the SRS signal of only a single molecular vibration rather than a full spectrum. Further, except for rare examples,^4^ it is dependent on detecting the signal transmitted through the sample rather than epi-detection of backscattered light. Recently, broadband SRS microscopy has been advanced by several laboratories, but these methods make epi-detection particularly difficult.^5^ Recent experiments have demonstrated that FSRS of solid-state samples could be made possible via epi-detection in a microscope.^6^ While these experiments extend the sample type from transparent liquids to include solid crystals, a more general method for opaque and turbid samples will advance the applicability of FSRS to more complicated systems.

By augmenting traditional FSRS collection optics, we collected the stimulated Raman spectra of opaque samples. Further, we are able to compare the intensity and polarization of the new diffuse reflectance-based femtosecond stimulated Raman spectroscopy (drFSRS) data with traditional FSRS data.

## Experimental Section

### Solutions

Ethanol (200 Proof, Koptec), cyclohexane (HPLC grade, Fisher Chemical), 10× phosphate buffered saline (PBS, Sigma), 20% Intralipid (Sigma), and poly(tetrafluoroethylene) (PTFE, 35 μm particle size, Sigma) were used without further purification.

#### Cyclohexane Solutions

To prepare the cyclohexane-intercalated PTFE solution, roughly 100 mg of PTFE was mixed with 10 mL of cyclohexane to create a slurry. A 1 mL aliquot of the slurry was then pipetted into a 2 mm cuvette (Starna) and stored upright for ca. 10 min. Once a stable pellet of PTFE was formed due to gravity, spectroscopic measurements of the pellet were taken. The pellet strongly scattered the incident probe beam so that there was no measurable transmitted power. PTFE solid films have been established as extremely strong, uniform scattering materials, with a mean free path of just 6 μm in solid films.^7^

#### Ethanol Solutions

To prepare the semiopaque ethanol solutions, a stock solution of 4% intralipid was made by dissolving 10 mL of 20% intralipid solution with 40 mL of PBS. One milliliter of 4% intralipid solution was then mixed with either 0, 0.5, or 1 mL of neat ethanol and 3, 2.5, or 2 mL of PBS to make 0%, 12.5%, and 25% by volume ethanol solutions, respectively, with 1% intralipid as the scattering agent. The solutions were then sonicated for ca. 3 min prior to being pipetted into a 1 mm cuvette (Starna) for spectroscopic measurement. The white suspension transmitted just 1.5% of the incident probe along the probe direction and scattered ca. 0.5% of the probe in the forward direction (within 20° of the transmitted probe) and backscattered ca. 0.3% of the power within a 1 cm^2^ area, displaced 10–20° from the incident beam. Intralipid suspensions have been previously characterized to model biological tissue, establishing that the mean-free path in a 1% suspension is 70 μm.^8^

### Optical Design

**Figure 1:**
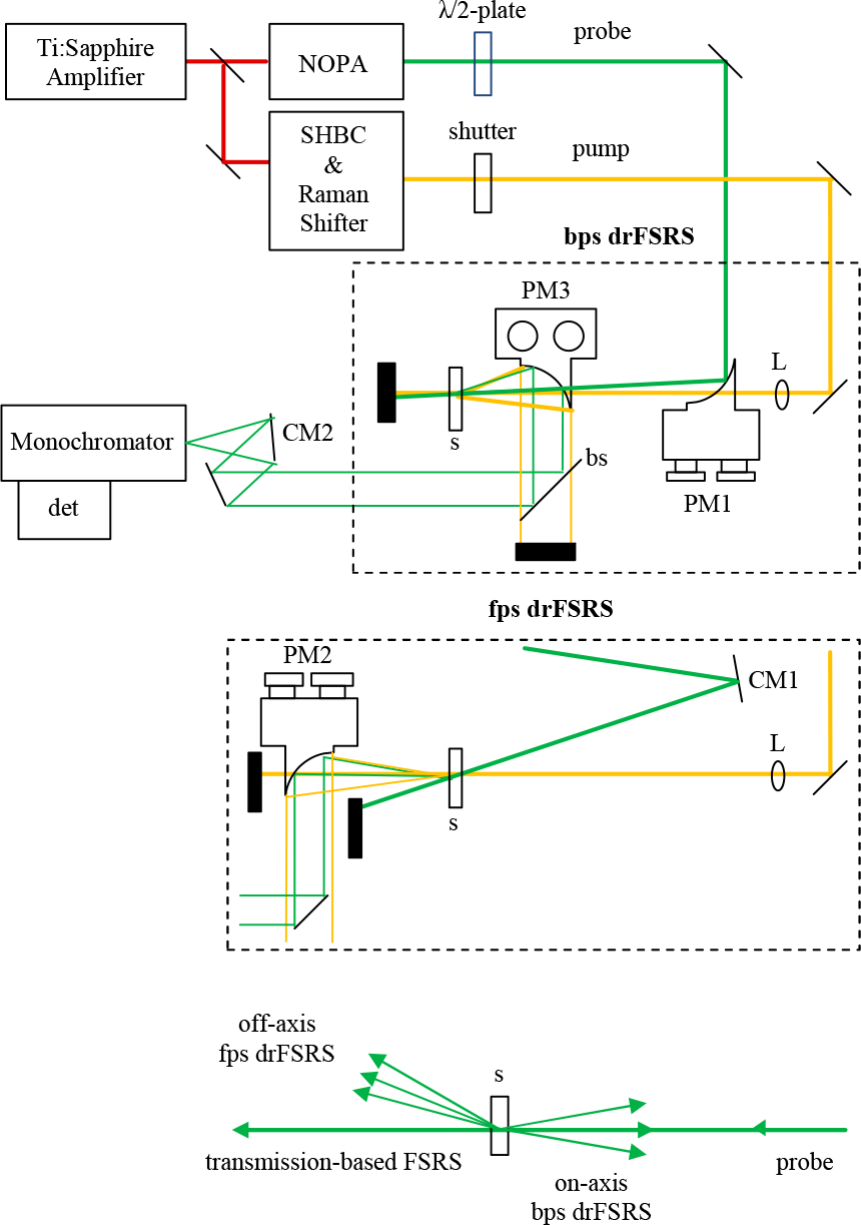
Laser and collection optics for bps drFSRS and fps drFSRS. PM = parabolic mirror with PM1, 4” efl ⌀ = 1.5”; PM2, 4” efl ⌀ = 1.5”; PM3, 1” efl ⌀ = 1”; L, 150 mm fl focusing lens; s, sample; CM1, 6” fl spherical curved mirror; CM2, 100 mm fl spherical curved mirror; bs, 594 nm long-pass dichroic beamsplitter; NOPA, noncolinear optical parametric amplifier; SHBC, second harmonic bandwidth compressor. At the bottom, we indicate which signal is collected in which direction.

Each FSRS or drFSRS experiment occurs by focusing pump and probe beans on the sample, temporally and spatially overlapped with beam diameters of 40–50 μm and crossing angles of 7–15°. Two novel collection methods of FSRS are presented in Figure 1: forward propagating scattered-probe diffuse reflectance-based FSRS (fps drFSRS) and backward propagating scattered-probe diffuse reflectance-based FSRS (bps drFSRS). Unless explicitly stated, drFSRS refers to bps drFSRS, in which the collected light has been scattered backward from the opaque solution. For clarity, fps drFSRS differs from standard transmission-based FSRS as fps drFSRS utilizes probe light that has transmitted through the sample but has optically scattered off the turbid sample and emerges from the back-side of the cuvette off-axis comparative to the nonscattered transmitted probe beam. In a fps drFSRS configuration, the on-axis transmitted, nonscattered probe photons are blocked as shown in Figure 1, although this beam is collected in normal transmission-based FSRS.

For fps drFSRS, a 4 in. effective focal length 90° off-axis ⌀ = 1.5-in. parabolic mirror (ThorLabs) was used to collect the transmitted probe light that was scattered off-axis. The collection efficiency for a 1.5 in. parabolic mirror 4 in. from the sample point is only 0.86%, but this mirror allowed for simple separation of the transmitted probe beam and the off-axis forward-propagating scattered probe light. Spatial separation of the transmitted probe and the forward-propagating scattered light allowed for the blocking of either component. For bps drFSRS, a 1 in. effective focal length 90° off-axis ⌀ = 1-in. parabolic mirror (Edmund Optics) was used to collect the backward propagating scattered probe pulse. The collection efficiency for this *f*/1 parabolic mirror is 5.28%. drFSRS requires efficient collection optics because of the need to collect as much probe light as possible, similar to spontaneous Raman. Hence, we have chosen to perform drFSRS with an efficient *f*/1 collection mirror in backscattering experiments. The pump and probe beams are focused through a hole in the middle of the parabolic mirror onto the sample and the collected bps drFSRS probe was then sent through a 594 nm long pass dichroic beam splitter (Semrock) to remove the Raman pump scatter.

### Laser Parameters

A commercial 800 nm 1 kHz femtosecond laser system (Spectra Physics Spitfire Pro) was used to generate both a short (ca. 50 fs) broadband probe pulse and a long (ca. 1.5 ps) narrowband pump pulse. The probe was of higher frequency (shorter wavelength) than the Raman pump, and an ultrafast Raman loss method of detection of the stimulated Raman signal was utilized.^9^ The broadband probe was generated using a noncollinear optical parametric amplifier (NOPA).^10^ The probe center was 559 nm with a fwhm of 25 nm and sufficient spectral tails to collect an SRS spectrum from 700 to 1700 cm^–1^. The Raman pump was generated by focusing the 400 nm output of a second harmonic bandwidth compressor^11^ through a 500 mm pipe filled with 68 atm of H_2_ gas.^12^ The second Stokes SRS transition of the H_2_ (600 nm with a fwhm of 16 cm^–1^) was then utilized as the Raman pump, resulting in a 16 cm^–1^ spectral resolution limit of the FSRS spectra

Full experimental details of the individual laser parameters used for transmission-based FSRS, fps drFSRS, and bps drFSRS can be found in the SI. Briefly, a NOPA probe ranging from 40 to 135 nJ/pulse and the Raman pump ranging from 1.7 to 4 μJ/pulse were used to generate stimulated Raman loss signal in the probe pulse. At these pulse energies, no sample damage occurred and no nonlinear optical distortion of either pulse was observed. The probe was then collimated using either transmissive or reflective collection optics and passed through a monochromator (Acton SpectroPro 2300i, 300 mm fl) with a 100 μm entrance slit and dispersed onto a CCD detector (PIXIS 100BR, Princeton Instruments). Spectra were collected using both LabView (National Instruments) and Winspec x32 (Princeton Instruments) and the SRS spectrum, SRS(*v*), is calculated in the usual manner as
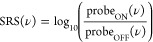
1in which probe_ON_(*ν*) is the probe spectrum collected with the Raman pump pulse on and probe_OFF_(*ν*) is the spectrum collected with the Raman pump blocked.

Note that, while it may be possible to perform spontaneous Raman spectroscopy with a similar optical system, it has been shown that the stimulated Raman signal exceeds the spontaneous signal by 7 orders of magnitude.^13^ However, rather than simply detecting these large numbers of photons, the challenge for any SRS experiment is to detect the modulation of the probe beam that is caused by the pump beam and occurs on top of noisy intensity fluctuations from the scattering sample. We have further reduced all spontaneous Raman scattering contributions by collecting in the short-wavelength region, where traditional anti-Stokes Raman signal would be detected. Any interfering anti-Stokes spontaneous Raman signal is further reduced by the negligible Boltzmann populations in this frequency region.

## Results and Discussion

### Cyclohexane:PTFE

**Figure 2:**
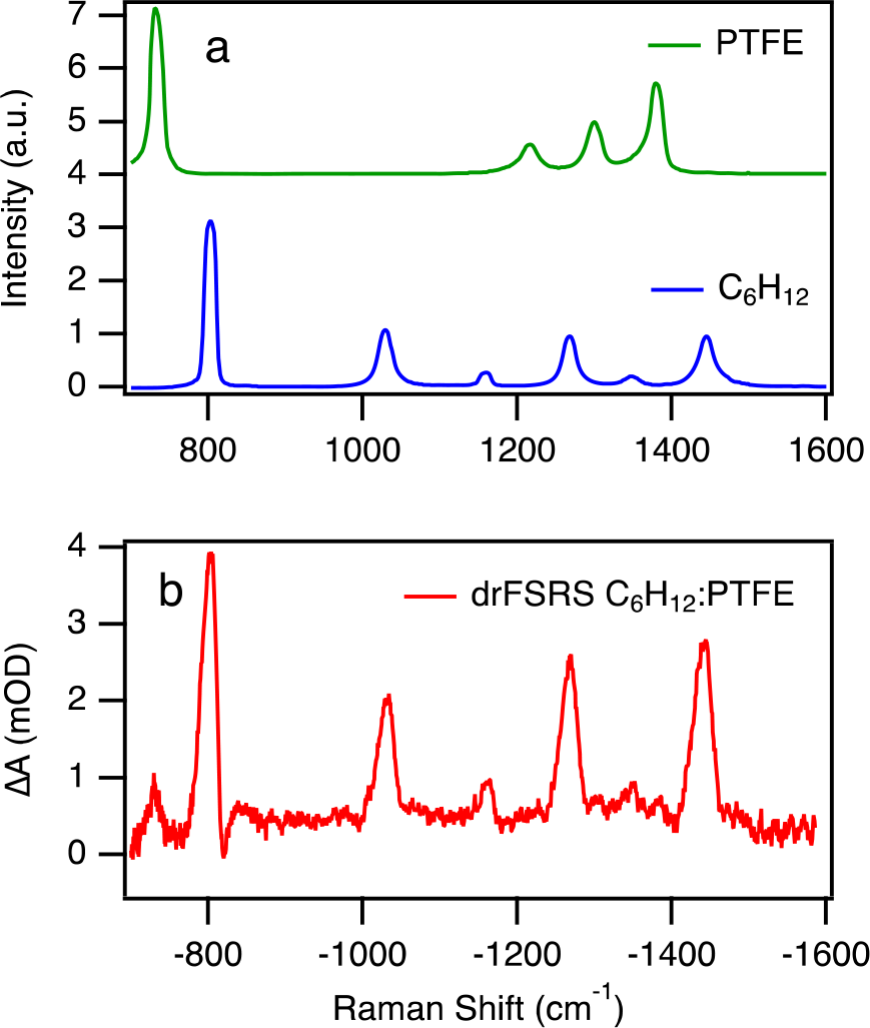
(a) Spontaneous CW Raman spectra of PTFE (green) and cyclohexane (blue) measured in the Stokes region. (b) bps drFSRS spectra of the cyclohexane:PTFE mixture measured as Raman loss SRS of the short-wavelength probe. drFSRS spectra are presented as collected, without baseline subtraction. Note that the negative frequencies in (b) are used to indicate SRS signals at frequencies higher than the Raman pump.

Bps drFRS was performed on 35 μm PTFE microbeads surrounded by cyclohexane. Figure 2a shows the spontaneous CW Raman spectra of cyclohexane and PTFE for comparison. Figure 2b shows the resulting drFSRS spectrum, collected in the short-wavelength region, which primarily resembles the Raman spectrum of cyclohexane. Upon closer inspection, however, Raman peaks associated with PTFE are observed at −729 and −1386 cm^–1^. These peaks indicate that both the target analyte (cyclohexane) and the scattering media (PTFE) undergo the SRS transitions that ultimately lead to signal generation.

When comparing the drFSRS spectrum of the mixture with a spontaneous Raman spectrum of the same mixture, both techniques measure the 1386 cm^–1^ peak height from PTFE to be about 15% the height of the 1446 cm^–1^ cyclohexane peak (Figure S1). However, the relative peak ratio between the 803 and 1029 cm^–1^ cyclohexane peaks is smaller in the drFSRS data than in the spontaneous Raman data, despite the similar instrumental spectral resolution. We believe that this is caused by a loss of polarization of the laser pulses as they interact with the scattering media.

**Figure 3:**
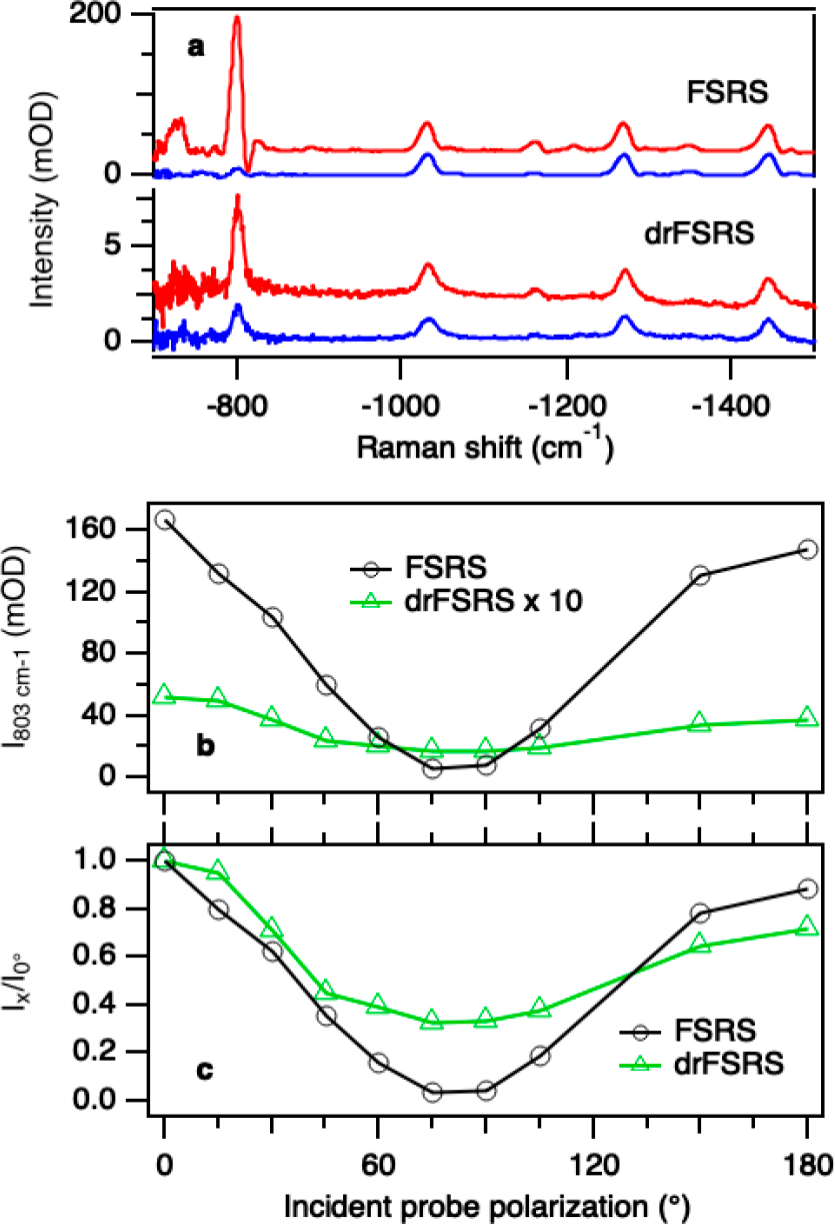
(a) FSRS of transparent cyclohexane and drFSRS of opaque cyclohexane:PTFE collected using 0° (red) and 90° (blue) incident probe polarization with respect to constant pump polarization. (b) The measured intensity of the 803 cm^–1^ cyclohexane peak height as a function of incident probe polarization using FSRS (black circles) and drFSRS (green triangles, x10 scaling). (c) Normalized intensity of the 803 cm^–1^ cyclohexane peak as a function of incident probe polarization using FSRS (black circles) and drFSRS (green triangles). In (a), the spectra are offset vertically for clarity. FSRS spectra in (a) have had a broad baseline subtracted; drFSRS spectra in (a) are presented without baseline subtraction.

The polarization dependence of signals is observed in Figures 3 and S4. The FSRS spectra of cyclohexane and drFSRS spectra of cyclohexane:PTFE taken at parallel and perpendicular pump–probe polarization are shown in Figure 3a. The 803 cm^–1^ drFSRS signal demonstrates a smaller loss of signal as a function of incident pump–probe polarization compared with its transparent FSRS counterpart. The 803 cm^–1^ peak of cyclohexane has been shown to exhibit a 0.04 depolarization ratio,^1,14^ thus its intensity is highly dependent on the relative polarization of the pump and probe. The depolarization of this peak as measured by using drFSRS is 0.32 (Figure 3c), whereas the depolarization of this peak as measured by using FSRS is 0.037. We expect that the increased depolarization ratio for drFSRS is due to the loss of polarization of the pump and probe beams as they scatter off particles in the suspension. Upon scattering, the incident pump–probe polarization is changed, which either increases (at near perpendicular pump–probe polarization) or decreases (at near parallel pump–probe polarization) the expected signal strength for a strongly polarized Raman peak. Curiously, this effect is only measurable for the highly polarized 803 cm^–1^ peak; the peaks at 1029, 1267, and 1446 cm^–1^, all should have depolarization ratios of 0.75, which is observed in both FSRS of transparent samples and the bps drFSRS of the opaque cyclohexane:PTFE mixture (Figure S4). For these modestly polarized bands, fluctuations in signal intensity between spectra make the small increase in signal due to depolarization of the beams difficult to measure. We expect that magic-angle polarization between the two pulses would produce FSRS and drFSRS spectra with equivalent relative intensities.

The cyclohexane drFSRS signal in the presence of the PTFE microbeads, 5 mOD, is significantly less than an equivalent path length transmissive FSRS signal of a transparent sample, 165 mOD. The loss of SRS signal in drFSRS is likely caused by a decreased concentration of cyclohexane molecules in the pump–probe interaction volume as the PTFE microbeads contribute a significant portion of matter located within the interaction volume. If the PTFE is treated as tightly packed nonporous spheres, the volume remaining within the interaction region remaining to cyclohexane would be 1 – π/3√2 (ca. 26%), as dictated by face-centered cubic packing. We would expect the signal strength then to decrease to as low as 41 mOD, 8 times greater than the measured signal strength. The remaining loss of signal strength must then come from losses of path length and pulse coherence.

### Ethanol

To directly compare signal strength between transmission-based FSRS and drFSRS, spectra using both techniques on semiopaque ethanol solutions were collected. 1% intralipid solutions were chosen to produce a suspension in which the probe beam would both transmit (for FSRS) and scatter the probe through (for fps drFSRS) and off (for bps drFSRS) the sample. Intralipid also has well characterized optical properties.^8^ A spontaneous Raman spectrum of a solution of 1% intralipid and 25% ethanol obtained using a 594 nm CW laser showed only Raman peaks from ethanol in the range of 700 to 2000 cm^–1^ (Figure S3).

**Figure 4:**
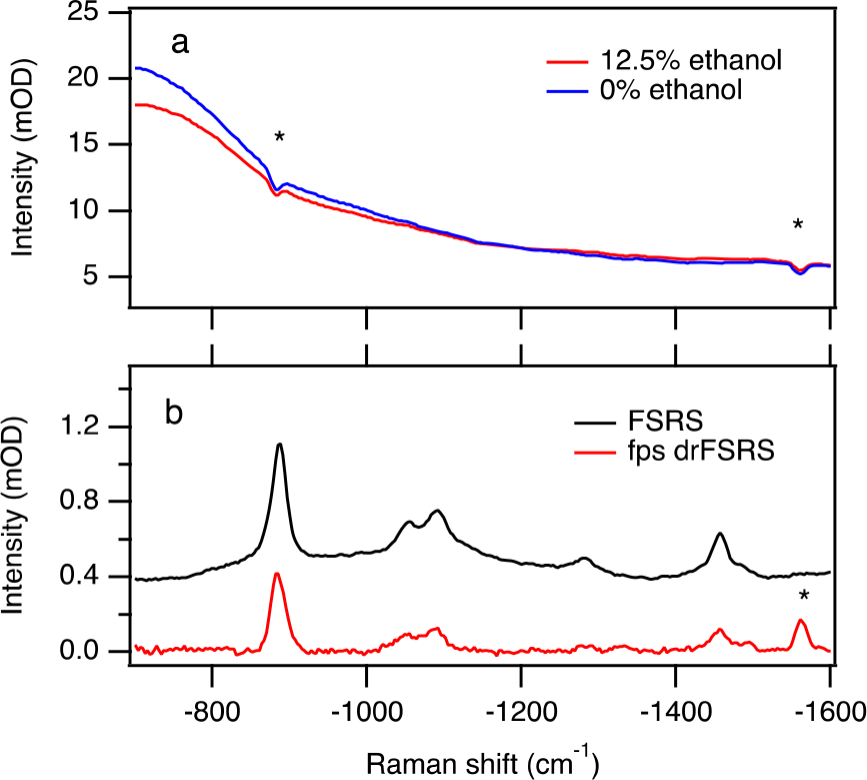
(a) Forward propagating scattered-probe drFSRS spectrum of 12.5% ethanol (red) and 0% ethanol (blue) in 1% intralipid solution. (b) Stimulated Raman spectra of 12.5% ethanol in 1% intralipid-containing solution collected using transmission-based FSRS (black, by collecting the transmitted probe light) and fps drFSRS (red, by collecting the off-axis forward scattered probe light), which is the difference between the spectra of panel a after an additional baseline subtraction step. In panel (a), two artifacts on the CCD are visible that are marked by asterisks (*). One of these artifacts remains in the difference spectra of panel (b) at −1561 cm^–1^.

Using a solution of 1% intralipid and 12.5% ethanol, a comparison of signal strength as obtained by transmission-based FSRS and fps drFSRS was made. The fps drFSRS spectrum obtained was the difference spectrum of a 12.5% ethanol in 1% intralipid solution and a 0% ethanol in 1% intralipid solution (Figure 4a). Further, a hand-drawn baseline was removed from the difference spectrum (Figure S6). As seen in Figure 4b, the 885 cm^–1^ ethanol peak height obtained using fps drFSRS (red) was 59% of that of the peak height obtained using transmission-based FSRS. Comparing the Raman spectra of 25% ethanol in 1% intralipid-containing solution measured using bps drFSRS to transmission-based FSRS, the 885 cm^–1^ ethanol peak height using drFSRS was 85% of that of the peak height obtained using transmission-based FSRS (Figure S7).

These spectra indicate that when the path lengths are comparable and the sample does not contain totally symmetric strongly polarized Raman peaks, drFSRS and traditional FSRS will obtain comparable signal strengths. Hence, the 8-fold loss of signal in the drFSRS of cyclohexane:PTFE sample compared to that from transparent cyclohexane must indicate that the opaque PTFE suspension limits the interaction path length of the pump and probe by a factor of 8, to just ca. 0.25 mm.

The ethanol features present in the transmission-based FSRS spectra are also present in the drFSRS spectra. While there is a loss of SRS signal strength when using drFSRS, the spectral resolution is unaffected. The fwhm of the 885 cm^–1^ was measured as 23.9 ± 0.4 and 22.4 ± 0.4 cm^–1^ using transmission-based FSRS and fps drFSRS, respectively.

## Conclusion

By implementing new collection optics, a diffuse reflectance-based FSRS was developed. The stimulated Raman spectra of both opaque and semiopaque solutions were measured. The opaque cyclohexane:PTFE bps drFSRS data demonstrated that both the scatterer (PTFE) and the analyte (cyclohexane) undergo the SRS process and significant depolarization of the incident beams occurs, distorting the measured depolarization ratio of the cyclohexane peaks. The drFSRS data of semiopaque ethanol solution demonstrated that there is a slight loss of signal strength when comparing drFSRS, both in fps and bps drFSRS, to FSRS, but there is no loss of spectral resolution. These results suggest that drFSRS could be used as a tool to measure the structural dynamics of fluorescent opaque samples as the data from drFSRS retain many of the same traits as the data obtained from traditional, transmissive FSRS experiments.

## Supporting Information Available

Explicit experimental parameters for FSRS, bps drFSRS, fps drFSRS, and spontaneous Raman spectroscopy; bps drFSRS and spontaneous Raman spectra of cyclohexane:PTFE; FSRS and bps drFSRS spectra of cyclohexane:PTFE collected using varying incident probe polarization; spontaneous Raman spectrum of ethanol in 1% intralipid solution; fps drFSRS spectrum of ethanol in 1% intralipid solution; FSRS and bps drFSRS spectra of ethanol in 1% intralipid solution (PDF)
