# Supplementary material for: Diffuse Reflectance-Based Femtosecond Stimulated Raman Spectroscopy of Opaque Suspensions
Source: Anal Chem. 2023 Oct 18;95(43):15856–60. doi: 10.1021/acs.analchem.3c02491 (PMC10620773; doi:10.1021/acs.analchem.3c02491)
Supplement: Supplementary file 1 — ac3c02491_si_001.pdf [file ac3c02491_si_001.pdf]

Supplementary Information for:
Diffuse Reflectance-Based Femtosecond Stimulated Raman Spectroscopy of Opaque Suspensions

Steven A. Diaz, David W. McCamant*

University of Rochester,
Department of Chemistry
Rochester, NY 14534 USA

Table of Content

**Laser parameters**	**Section 1**
**Additional spectra used in interpretation of bps drFSRS**	**Section 2**
bps drFSRS and CW Raman spectra of cyclohexane:PTFE	Figure S1
Transmission-based FSRS spectra of cyclohexane taken at varied incident probe polarization	Figure S2
bps drFSRS spectra of cyclohexane:PTFE taken at varied incident probe polarization	Figure S3
Polarization dependent intensity of FSRS and drFSRS peaks	Figure S4
**Additional spectra used in interpretation of fps drFSRS**	**Section 3**
Spontaneous Raman spectrum of 25% by volume ethanol in 1% Intralipid	Figure S5
fps drFSRS spectrum of 12.5% ethanol in 1% Intralipid	Figure S6
Transmission-based and bps drFSRS spectra of 25% ethanol in 1% Intralipid	Figure S7

Section 1. Laser parameters

Backward Propagating drFSRS. For the cyclohexane-intercalated PTFE measurements, 40 nJ/pulse at 555 nm was used as the probe pulse, and 1.7 µJ/pulse at 600 nm was used for the pump pulse. A monochromator with a 100 µm entrance slit and an 1800 grooves/mm grating (Acton SpectroPro 2300i, 300 mm fl) was used to disperse the probe onto a CCD detector (PIXIS 100BR, Princeton Instruments).

The cyclohexane-intercalated PTFE drFSRS data was collected using home-built LabView software (National Instruments). Each pump-on and pump-off probe spectrum was collected using a 400 ms exposure time and averaged 40 times. To further increase the signal-to-noise ratio, these averaged spectra were collected a total of 51 times for a total of 27.2 minutes worth of exposure time.

For the semi-opaque ethanol solution measurements, a 135 nJ 563 nm pulse was used as the probe, and a 2.5 µJ 600 nm pulse was the pump. The probe was then passed to the monochromator with a 600 grooves/mm grating and detected on the aforementioned CCD.

The semi-opaque ethanol drFSRS spectrum was collected using Winspec x32 (Princeton Instruments). Each pump-on and pump-off probe spectrum was collected using a 30 second total exposure time (600 accumulations of 50 ms exposures to prevent signal saturation) and averaged 4 times. The resulting spectra were collected using a total of 4 minutes worth of exposure time.

Forward Propagating drFSRS. For the semi-opaque ethanol solution measurements, the probe was 110 nJ/pulse at 563 nm, and the pump was 4 µJ/pulse at 600 nm. The probe was dispersed with a 600 grooves/mm grating and detected using the CCD detector. Transmissive-based FSRS were performed on the semi-opaque ethanol using the same laser parameters.

The semi-opaque ethanol fps drFSRS spectrum was collected using a scanning multichannel technique (SMT) in LabView.[1] Briefly, the fps drFSRS spectrum was the result of an average of sixty-four 21-point scans, each consisting of 1000 pump-on/pump-off averages per point or a total of 44.8 minutes. The transmissive-based FSRS spectrum was the result of an average of thirty-two 21-point scans consisting of 1000 averages per point or a total of 22.4 minutes.

Transmission-based FSRS. For the transparent neat cyclohexane measurements, the probe was 40 nJ/pulse at 555 nm and the pump was 1.7 µJ/pulse at 600 nm. The transmitted-and-not-scattered probe beam was collected and collimated and passed to the monochromator (Acton). The entrance slit to the monochromator was set at 100 µm, and an 1800 grooves/mm grating was used to disperse the probe onto the CCD. The transparent cyclohexane FSRS data was collected using LabView. Each spectrum was the average of 1000 1-ms exposure time or a total of 2 seconds.

Spontaneous (CW) Raman. The spontaneous Raman spectra of PTFE, cyclohexane, cyclohexane:PTFE, and ethanol solutions were collected by focusing 70 mW of a 594 nm continuous wave (CW) laser (Cobolt) onto the sample. The Raman scattering was then collected and collimated, passed through a 594 nm notch filter (StopLine, Semrock), and sent through a single stage of a triple monochromator (TriVista TR555, Princeton Instruments). The Raman signal was then imaged onto a CCD array (PIXIS 400BRX, Princeton Instruments).

Section 2. Additional spectra used in the interpretation of bps drFSRS

The cyclohexane:PTFE system used in backward propagating scattered-probe diffuse reflectance-based FSRS (bps drFSRS) has little-to-no fluorescence when probed using spontaneous Raman. In Figure S1, we show the bps drFSRS collected in the anti-Stokes region and the CW Raman spectra of cyclohexane:PTFE collected in the Stokes region. Note that in FSRS, the "anti-Stokes" region is where stimulated Raman loss is observed from known coherent Raman pathways; it is not due to a traditional anti-Stokes Raman transition from v=1 to v=0. Hence, the relative intensities in (a) and (b) are expected to be the same and unaffected by thermal population of excited vibrational states.

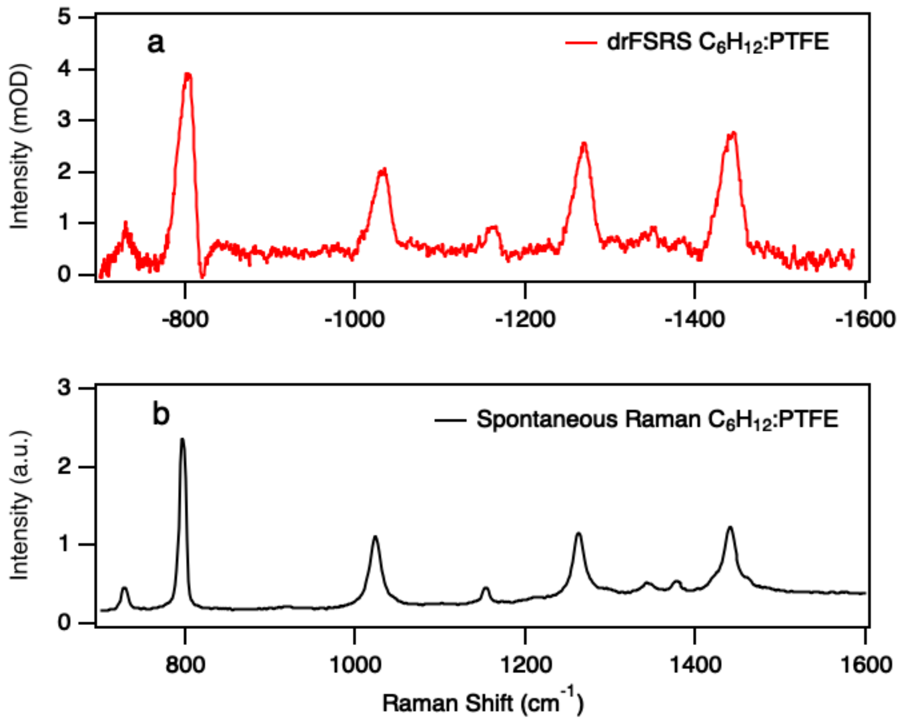

Figure S1. (a) bps drFSRS spectrum of cyclohexane-intercalated PTFE beads (red) measured in the anti-Stokes region (-700 to -1600 cm^{-1}). (b) Spontaneous Raman spectrum of cyclohexane-intercalated PTFE beads (black) measured in the Stokes region (700 to 1600 cm^{-1}). The data is presented without the removal of any broad baseline artifacts.

Large underlying baseline artifacts were present when collecting the transmission-based FSRS of neat cyclohexane (Figure S2), likely due to standard cross-phase modulation effects often observed in FSRS.[2] To best observe the intensity of the FSRS data as a function of incident probe polarization (with constant pump polarization), a hand-drawn baseline was removed from the FSRS data. These underlying baseline artifacts were not present when collecting the bps drFSRS of cyclohexane:PTFE (Figure S3).

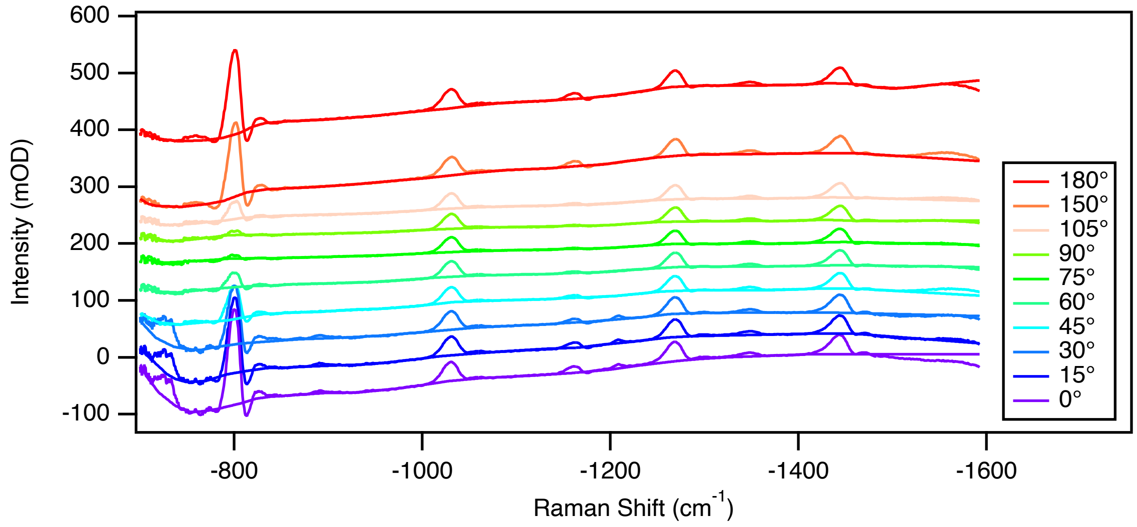

Figure S2. Transmission-based FSRS spectra of neat cyclohexane using varied incident probe polarization with drawn baselines. Spectra are shifted vertically for clarity. Displayed baselines are drawn in and subtracted to remove the cross-phase modulation background.[2]

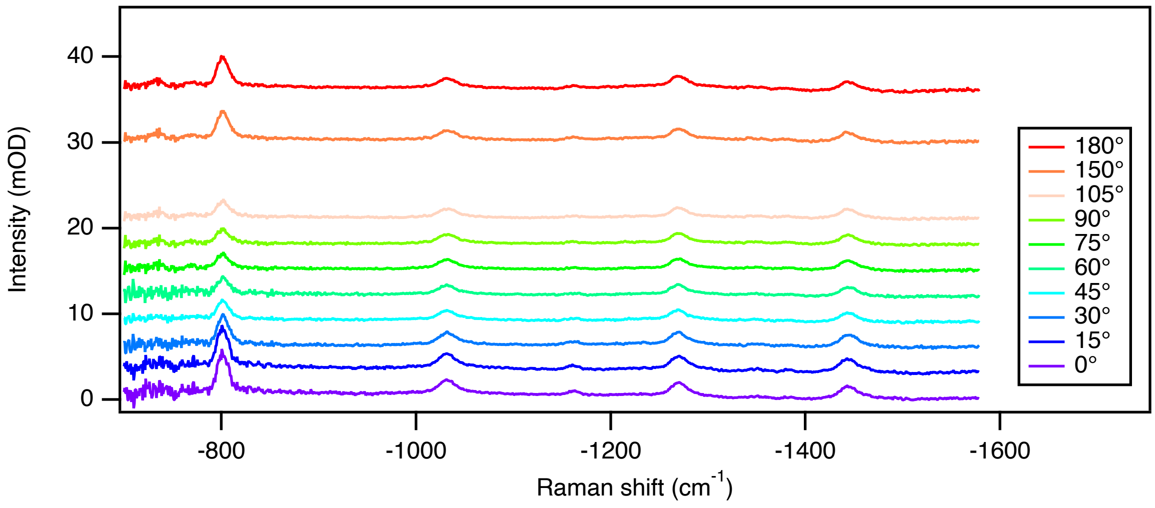

Figure S3. bps drFSRS spectra of cyclohexane:PTFE collected using varied incident probe polarization. Data is presented as collected, without any baseline drawing and subtraction, though spectra are offset vertically for clarity.

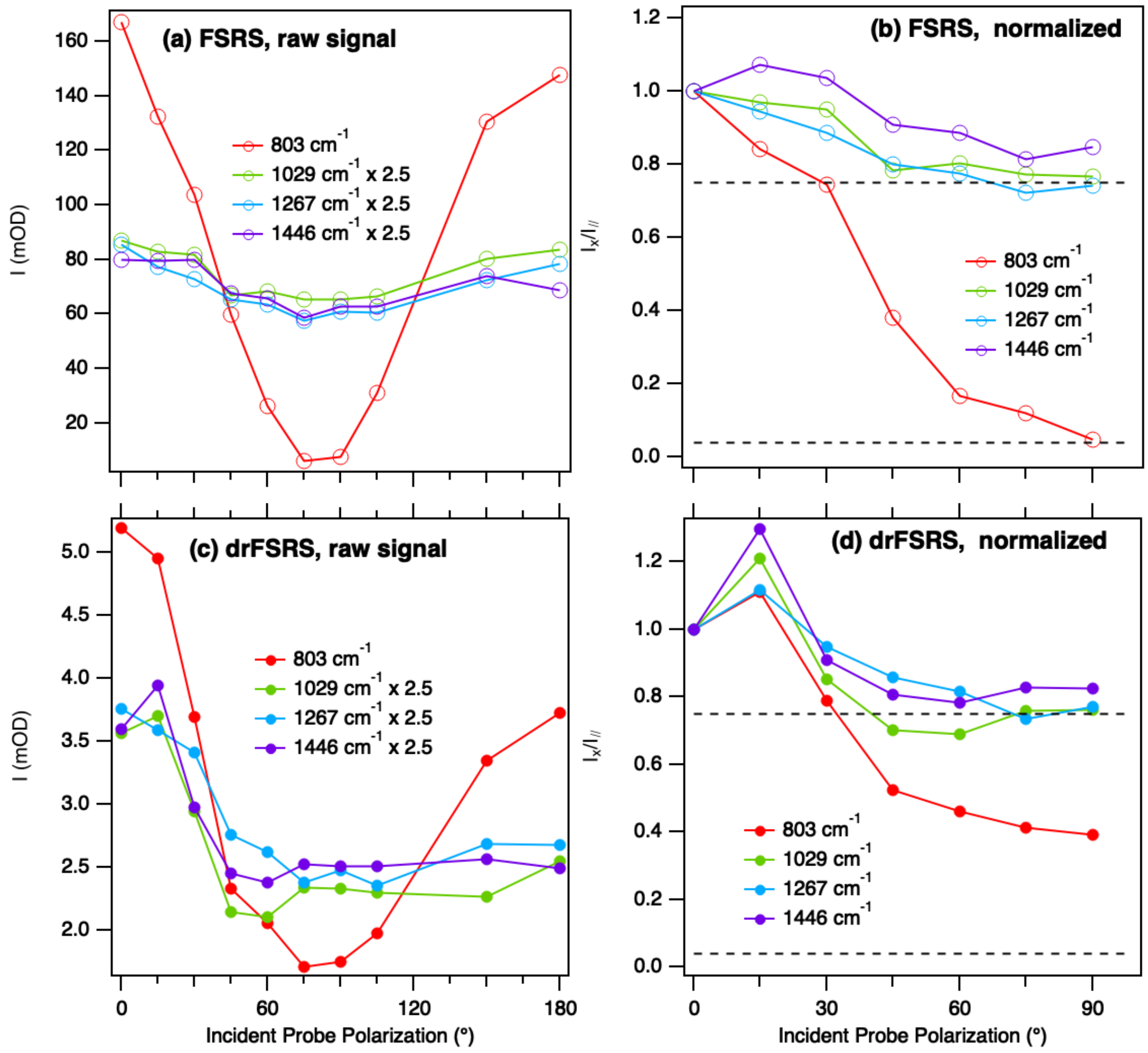

Figure S4. a) The measured intensity of the 803 cm^{-1} (red), 1029 cm^{-1} (green), 1267 cm^{-1} (blue), and 1446 cm^{-1} (purple) stimulated Raman signal of neat cyclohexane as a function of incident probe polarization collected using transmission-based FSRS. b) The normalized intensity of the 803 cm^{-1} (red), 1029 cm^{-1} (green), 1267 cm^{-1} (blue), and 1446 cm^{-1} (purple) stimulated Raman signal of neat cyclohexane as a function of incident probe polarization collected using transmission-based FSRS. c) The measured intensity of the 803 cm^{-1} (red), 1029 cm^{-1} (green), 1267 cm^{-1} (blue), and 1446 cm^{-1} (purple) stimulated Raman signal of cyclohexane-intercalated PTFE as a function of incident probe polarization collected using bps drFSRS. d) The normalized intensity of the bps drFSRS signals of cyclohexane-intercalated PTFE as a function of incident probe polarization. The dashed lines in panels b and d are at 0.04 and 0.75, the expected depolarization ratios of the various peaks. The depolarization ratio of 803 cm-1 peak is expected to be 0.04 and the 1029, 1267 and 1446 cm^{-1} modes are all expected to be 0.75 in a traditional Raman experiment.

In the transparent sample, the polarization of the FSRS signals from cyclohexane behave as expected (Fig. S4 a and b), but when collected using bps drFSRS the depolarization ratio of the 803 cm^{-1} peak is much higher than the expected 0.04. The weaker peaks in the spectrum at 1029, 1267 and 1446 cm-1 are all expected to have a 0.75 depolarization ratio, which is accurately displayed in the FSRS spectrum of the transparent sample. In the bps drFSRS experiment, these peaks continue to display similar depolarization ratios of about 0.75-0.80, despite the large scattering induced by the PTFE.

Section 3. Additional spectra used in the interpretation of fps drFSRS.

To ensure no vibrational Intralipid features were present in the region of interest for fps drFSRS of ethanol, CW Raman of 25% by volume ethanol in 1% Intralipid was collected (**Figure S5**). All the features present in this spectrum belong to ethanol.

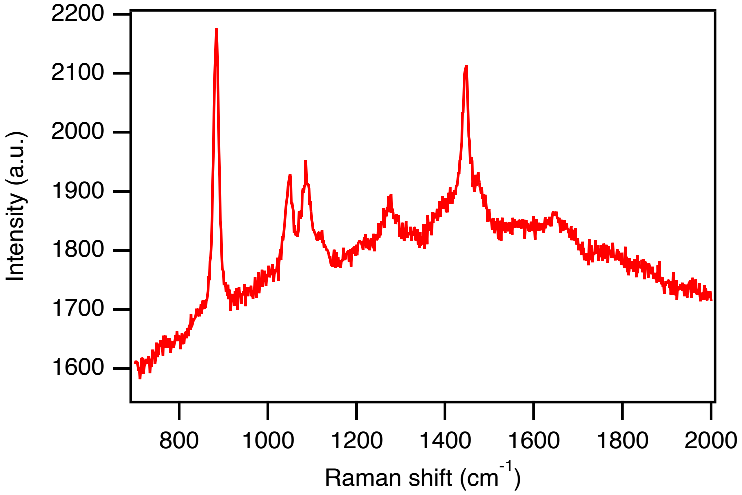

Figure S5. Spontaneous Raman spectrum of 25% by volume ethanol in 1% Intralipid using 70 mW of 594 nm excitation laser. The data were the average of three 20 second exposures and are presented without any baseline subtraction.

To measure the fps drFSRS spectrum of 12.5% by volume ethanol in 1% Intralipid-containing solution, a difference spectrum between the fps drFSRS data collected from 12.5 %v/v ethanol and 0 %v/v ethanol was performed. The data as collected are presented in Figure 4a in the main text. Upon subtraction, the spectrum shown in Figure S5 is obtained. This spectrum contains a large underlying baseline which was removed for Figure 4b in the main text with a hand-drawn baseline.

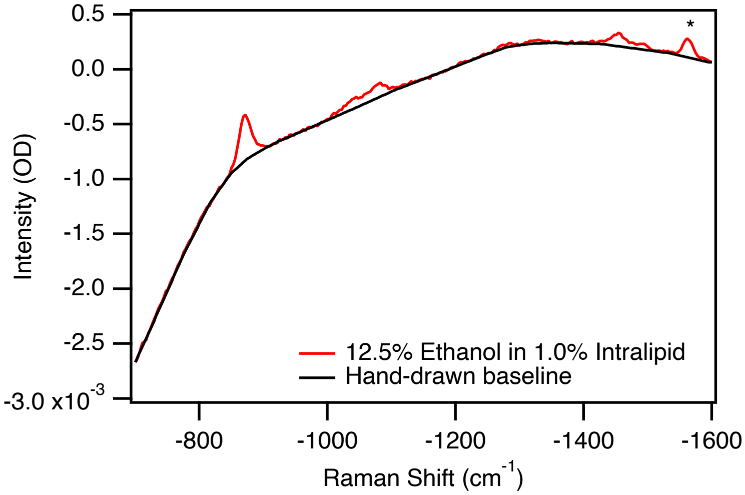

Figure S6. Solvent-corrected fps drFSRS spectrum of 12.5% ethanol with drawn baseline. The red spectrum is the difference between the 12.5% ethanol sample and the 0% ethanol sample, shown in Figure 4a. The spectrum shown in Figure 4b is the difference between this spectrum (red) and the baseline (black).

bps drFSRS of 25%v/v ethanol in 1% Intralipid-containing solution was also collected (Figure S6). The large underlying baseline in both the bps FSRS and transmission-based FSRS hides many of the expected features. While a hand-drawn baseline could be removed, the accuracy of the removed artifact would be low due to its intensity and odd shape.

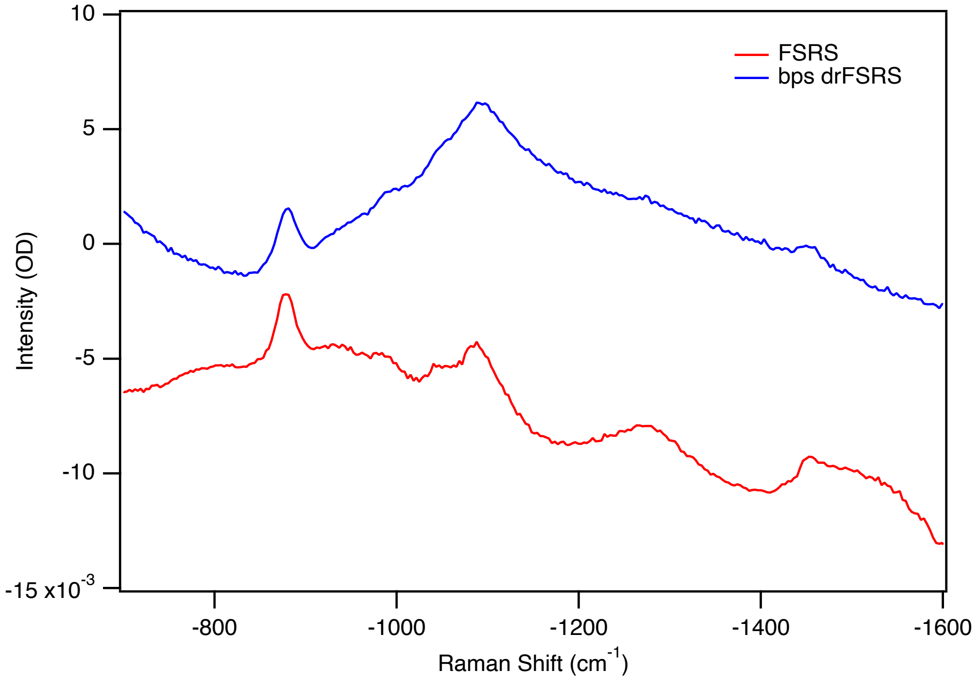

Figure S7. Stimulated Raman spectra of 25% ethanol in 1% Intralipid-containing solution collected using transmission-based FSRS (red) and backward propagating drFSRS (blue). The data are presented without the removal of baseline artifacts.

References

(1) Challa, J. R.; Du, Y.; McCamant, D. W. Femtosecond Stimulated Raman Spectroscopy Using a Scanning Multichannel Technique. *Applied Spectroscopy* **2012**, *66* (2), 227-232. DOI: 10.1366/11-06457.
(2) Würthwein, T.; Lüpken, N. M.; Irwin, N.; Fallnich, C. Mitigating cross-phase modulation artifacts in femtosecond stimulated Raman scattering. *J. Raman Spectrosc.* **2020**, *51* (11), 2265-2271. DOI: https://doi.org/10.1002/jrs.5958.
